# β_2_ Integrins As Regulators of Dendritic Cell, Monocyte, and Macrophage Function

**DOI:** 10.3389/fimmu.2017.01866

**Published:** 2017-12-20

**Authors:** Leonie Schittenhelm, Catharien M. Hilkens, Vicky L. Morrison

**Affiliations:** ^1^Institute of Infection, Immunity & Inflammation, University of Glasgow, Glasgow, United Kingdom; ^2^Institute of Cellular Medicine, Newcastle University, Newcastle upon Tyne, United Kingdom; ^3^Arthritis Research UK Rheumatoid Arthritis Pathogenesis Centre of Excellence (RACE), Glasgow, United Kingdom

**Keywords:** β_2_ integrins, CD11/CD18, dendritic cells monocytes and macrophages, immune regulation, autoimmunity

## Abstract

Emerging evidence suggests that the β_2_ integrin family of adhesion molecules have an important role in suppressing immune activation and inflammation. β_2_ integrins are important adhesion and signaling molecules that are exclusively expressed on leukocytes. The four β_2_ integrins (CD11a, CD11b, CD11c, and CD11d paired with the β_2_ chain CD18) play important roles in regulating three key aspects of immune cell function: recruitment to sites of inflammation; cell–cell contact formation; and downstream effects on cellular signaling. Through these three processes, β_2_ integrins both contribute to and regulate immune responses. This review explores the pro- and anti-inflammatory effects of β_2_ integrins in monocytes, macrophages, and dendritic cells and how they influence the outcome of immune responses. We furthermore discuss how imbalances in β_2_ integrin function can have far-reaching effects on mounting appropriate immune responses, potentially influencing the development and progression of autoimmune and inflammatory diseases. Therapeutic targeting of β_2_ integrins, therefore, holds enormous potential in exploring treatment options for a variety of inflammatory conditions.

## Introduction

The integrin family of proteins is comprised of 24 heterodimeric transmembrane adhesion receptors. Each integrin is formed through the non-covalent association of 1 α-subunit and 1 β-subunit; currently, 16 α-subunits and 8 β-subunits have been identified. Their expression on virtually all human cells and their complex signaling mechanisms explain their wide variety of biological roles, including blood clotting, cell adhesion, and migration.

**Figure 1:**
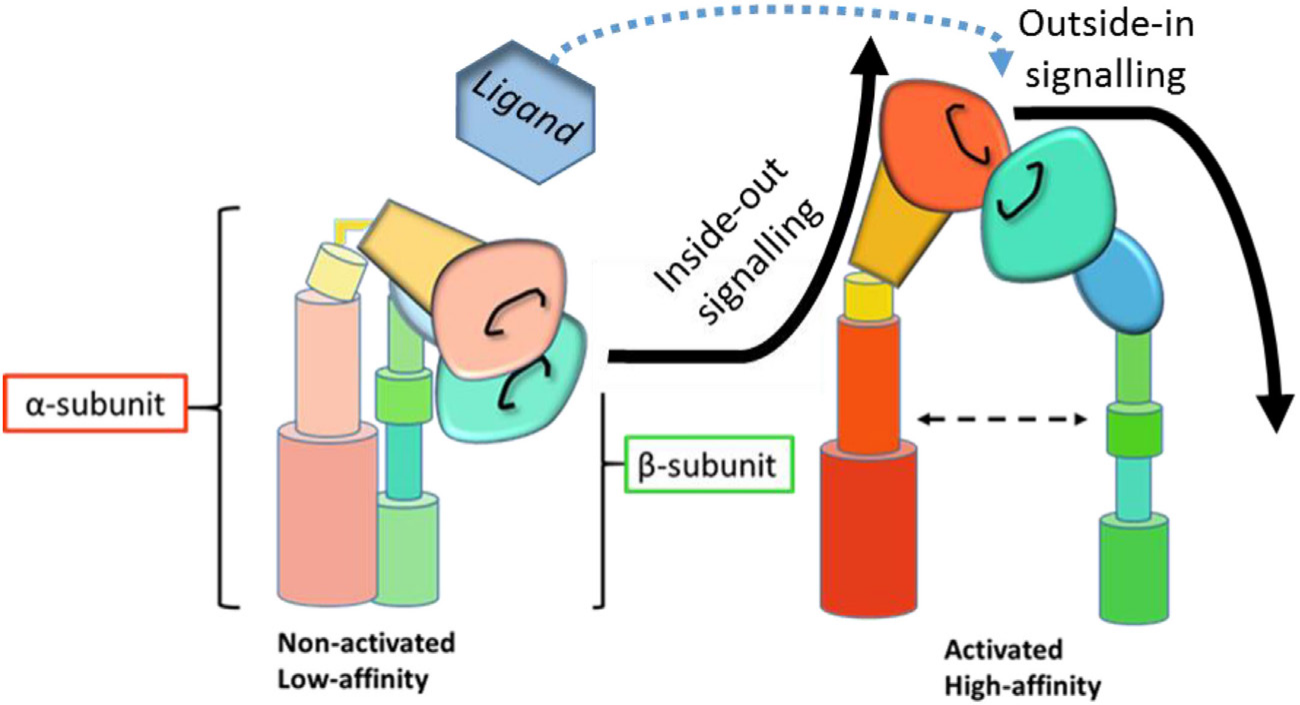
Schematic representation of integrin activation and signaling. Inside-out signaling induces a conformational change in the integrin to the active, high affinity state. Upon ligand binding, active integrins then transmit outside-in signals and downstream signaling cascades. [Adapted from Byron et al. (3), with permission from the *Journal of Cell Science*].

Due to their extensive importance in biological systems, elucidating integrin signaling and receptor function has been of great interest since their characterization as adhesion molecules over 30 years ago. Integrins are important signaling proteins that mediate interactions of the cell with extracellular matrix proteins and with other cells via cell-surface ligands. Integrins exist in a continuum between a folded inactive form with low affinity for their ligand and an extended high affinity conformation (1), although even bent integrins are able to bind ligand in rare instances (2). As immune cell adhesion and extravasation into lymph nodes and tissues forms part of initiating an effective immune response, β_2_ integrin conformation on the surface of leukocytes needs to be tightly regulated. β_2_ integrins on the surface of circulating leukocytes tend, therefore, to be largely inactive (2) until inside-out and outside-in signaling trigger integrin-mediated adhesion and extravasation into tissue (Figure 1).

Inside-out signaling modifies how cells interact *with* their environment by facilitating receptor affinity and avidity (4) to allow binding to extracellular ligands. Outside-in signaling, on the other hand, mediates intracellular events in response *to* their environment by eliciting downstream signaling cascades in response to receptor occupation. The complex details of integrin signaling are reviewed elsewhere (5, 6) and are beyond the scope of this review. Briefly, inside-out signaling is mediated by talin (7) and kindlin (8, 9) binding to the intracellular domain of the β_2_ subunit, a process initiated by chemokine receptor or Toll-like receptor (TLR) engagement (10, 11), which results in a conformational change in the integrin from a low-affinity to a high-affinity state. Outside-in signaling is then initiated by ligand binding to high-affinity integrin receptors (Figure 1). Downstream signaling events mediate the formation of focal complexes and adhesions through rearrangement of the actin cytoskeleton. The relative importance of affinity and avidity on integrin signaling and function is heavily debated (12, 13), but dynamic interaction between these processes and both inside-out and outside-in signaling seems likely (14).

**Figure 2:**
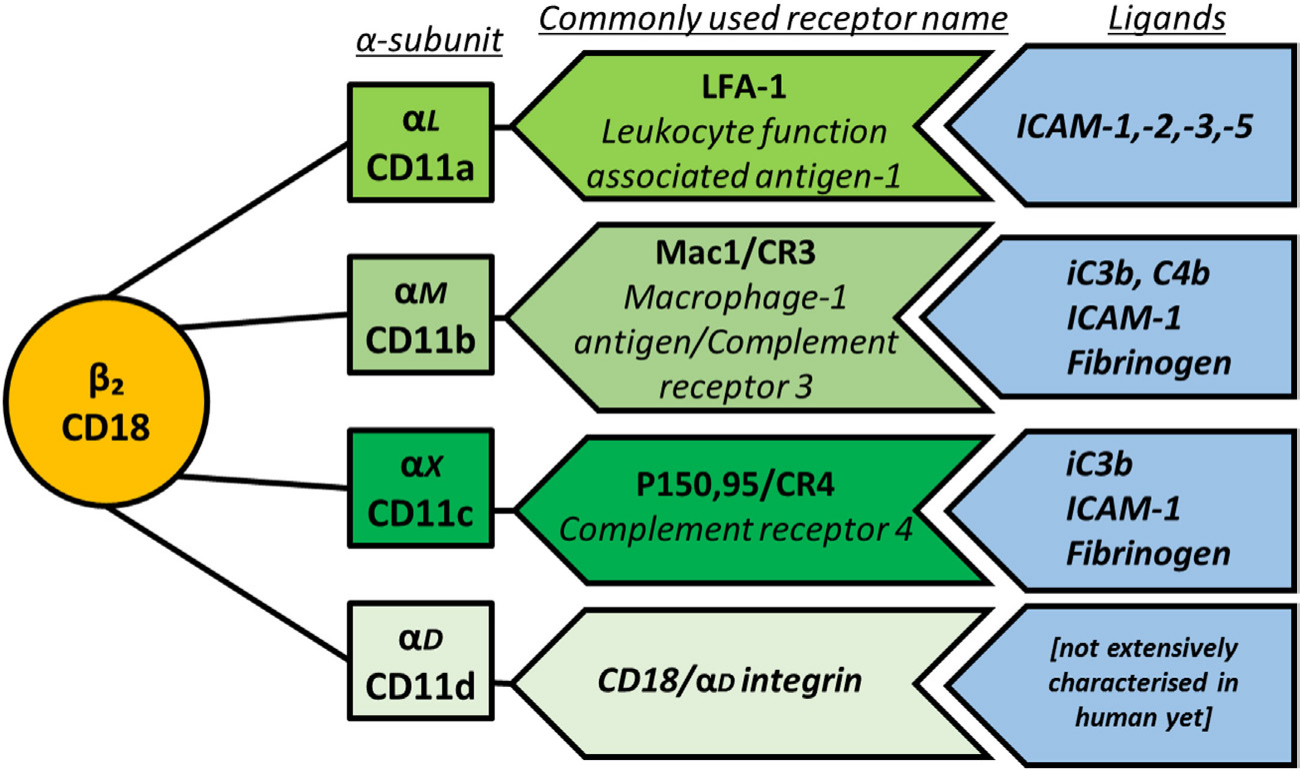
Schematic representation of β_2_ integrin subunit pairing, depicting the β-subunit CD18 as the common subunit non-covalently associating with one of four α-subunits. The main ligands for each integrin are also shown.

β_2_ integrins are the focus of this review, as they are exclusively found on leukocytes and therefore of particular importance for the immune system. They mediate cell recruitment into lymphoid organs and inflamed tissues by facilitating firm leukocyte arrest on endothelial cells and extravasation after cell rolling (15); cellular interactions between leukocytes including immunological synapse formation (16); and intracellular signaling cascades that influence cytoskeletal rearrangement, activation, proliferation and impact on cellular responses to TLRs. Importantly, through these three processes, β_2_ integrins can have either pro-inflammatory or anti-inflammatory outcomes. The β_2_ integrin subunit (CD18) can pair with one of four α-subunits (α_L_—CD11a, α_M_—CD11b, α_X_—CD11c, and α_D_—CD11d), forming leukocyte function-associated antigen-1, Mac1/CR3 (macrophage-1 antigen, complement receptor 3), P150,95/CR4 (complement receptor 4), and CD18/CD11d, respectively (Figure 2). For consistency, this review will utilize only the CD nomenclature. Both function and cell-specific expression of β_2_ integrins vary according to the α-subunit involved.

The main ligands for the β_2_ integrin family members are outlined in Figure 2. Briefly, CD11a binds to intracellular adhesion molecule-1 (ICAM-1), -2, -3, and -5, which are expressed by a variety of cells including leukocytes and endothelial cells, thereby mediating leukocyte recruitment to lymph nodes and sites of inflammation as well as cell–cell adhesion. CD11b binds the complement proteins iC3b and C4b with high affinity, mediating phagocytosis of complement-coated particles but can also bind ICAM-1, fibrinogen, and more than 40 other ligands (17). The sequence of CD11c is very close to that of CD11b, and indeed CD11c binds several of the same ligands including iC3b, ICAM-1, and fibrinogen. Multi-ligand binding capacity of CD11d is proposed to largely overlap with CD11b and includes ECM-associated proteins fibronectin, fibrinogen, vitronectin, Cyr61, and plasminogen (18).

**Figure 3:**
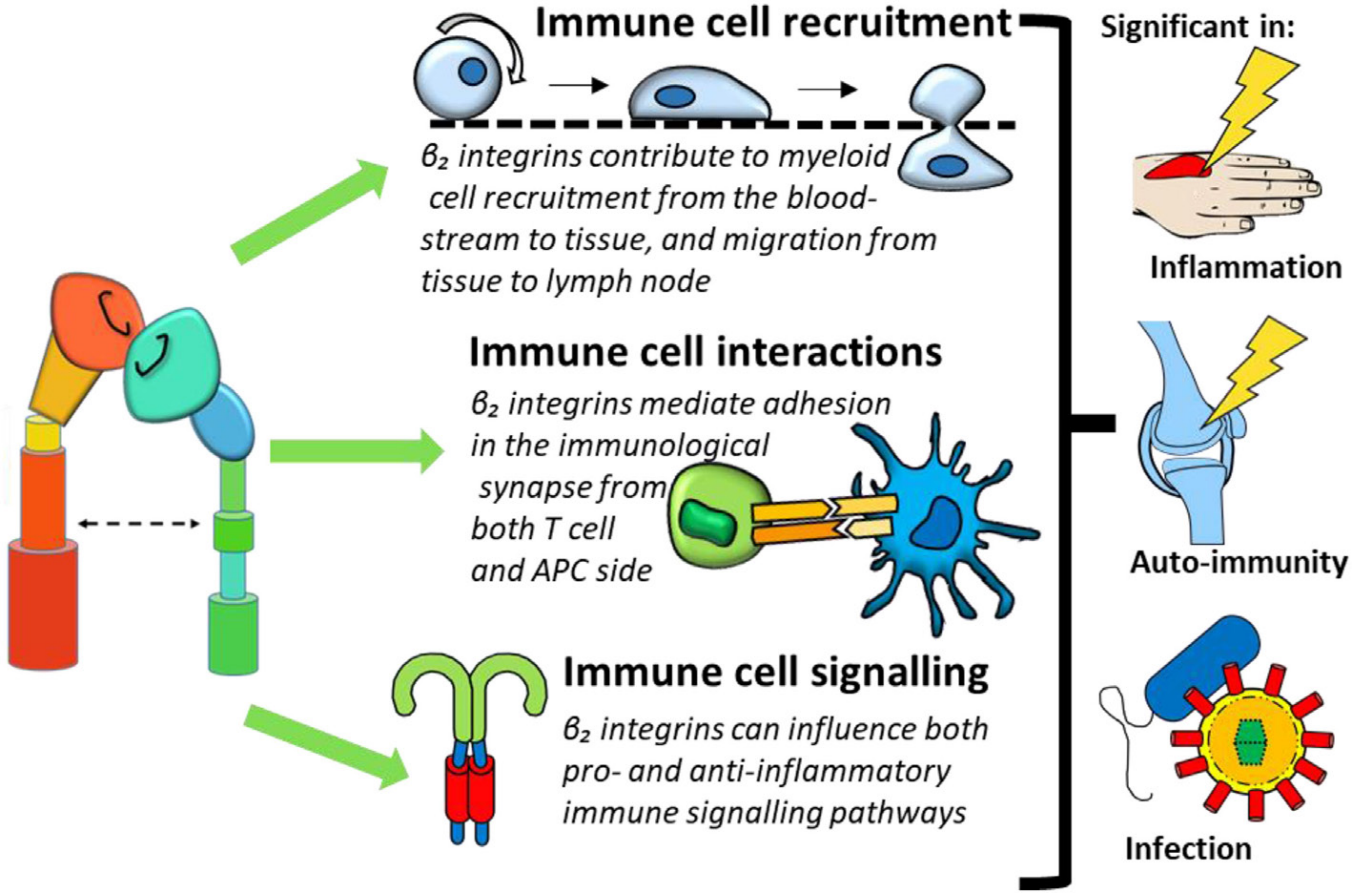
β_2_ integrin involvement in immune cell function can be categorized into three processes: immune cell recruitment, immune cell interactions, and immune cell signaling. Dysregulation of these functions could contribute to conditions such as inflammation, immunity, and infection.

This review will provide an overview of β_2_ integrin expression on monocytes, macrophages and DCs, before exploring the paradoxical pro-inflammatory and regulatory roles of β_2_ integrins in immune regulation in three key aspects of immune function: recruitment and migration, cellular interactions, and downstream cell signaling (Figure 3). We will furthermore review how dysregulated integrin signaling could contribute to inflammatory and autoimmune conditions and introduce the therapeutic potential of targeting β_2_ integrins.

## Expression of β_2_ Integrin Subunits by Dendritic Cells (DCs), Monocytes, and Macrophages

The expression of β_2_ integrin subunits varies in different leukocyte subsets and between mice and humans. In general terms, CD11a is expressed on all leukocytes at varying levels, while CD11b, CD11c, and CD11d are predominantly expressed by monocytes, macrophages and DCs. Specifically, in humans, monocytes express all four β_2_ integrin-associated alpha subunits (CD11a, CD11b, CD11c, and CD11d) with CD11a and CD11b expression greater than CD11c (19, 20); macrophages express CD11a and CD11b at lower levels than monocytes together with CD11c at similar levels to monocytes (21); while DCs mainly express CD11c together with CD11a, though some DC subsets also express CD11b (22). While CD11d has received less attention than the other β_2_ integrins due to the absence of commercially available human antibodies, Miyazaki and colleagues showed CD11d expression on monocyte-derived DCs and macrophages as well as most circulating monocytes (23). To complement the scarce available data, mRNA expression data for the CD11d subunit ITGAD were consulted. While Villani and colleagues (24) find monocytes to express highest levels of ITGAD mRNA, the Expression Atlas (25) reports highest expression in DCs, with ITGAD expression in monocytes remaining below detectable threshold. However, overall both RNAseq data sets show that CD11d mRNA expression is very low in monocytes, macrophages, and DCs. Table 1 provides the details of expression of all β_2_ integrin subunits in human and murine monocytes, macrophages, and DCs. Where available, expression analysis on DC subsets is given using the Guilliams nomenclature (26), which was recently confirmed and expanded by Villani and colleagues (24).

**Table 1:** β_2_ integrin expression on dendritic cells (DCs), monocytes and macrophages—human and murine findings.

Cell type	CD11a/CD18 (α_L_/β_2_)	CD11b/CD18 (α_M_/β_2_)	CD11c/CD18 (α_X_/β_2_)	CD11d/CD18 (α_D_/β_2_)
DCs	*Human*: high levels of CD11a on monocyte-derived DCs (22, 27–29); plasmacytoid DCs (pDCs) also express CD11a (30); reduced CD11a/CD18 levels upon DC activation (31)	*Human*: CD11b present on monocyte-derived DCs (22, 27, 28); detected in cDCs, but not in pDCs (31–33); higher on cDC2 than cDC1s (33, 34); reduced CD11b/CD18 levels upon DC activation (31)	*Human*: pDCs lack CD11c (31); expressed on mature DCs (31); CD11c expression is higher on cDC2 than cDC1s (33, 34); monocyte-derived DCs also express CD11c (32); reduced CD11c/CD18 levels upon DC activation (31)	*Human*: expressed on monocyte-derived DCs (23), single-cell mRNA data suggests low gene expression in DCs (25)

	*Mouse*: expressed by cDCs, particularly the CD8^+^ subset, and by pDCs (35); also highly expressed by bone marrow-derived DCs	*Mouse*: expression of CD11b in mouse cDCs is subset-specific: higher on CD8^−^ than CD8^+^ splenic DCs (35); expressed in sub-populations of gut DCs (36); absent from pDCs (37); expressed by bone marrow-derived DCs (38)	*Mouse*: CD11c highly expressed on cDCs and typically used as a DC marker (38); expressed by pDCs (39) and bone marrow-derived DCs (40)	*Mouse*: no protein expression data available, RNA-seq data suggest medium ITGAD gene expression in murine DCs (25)

Monocytes	*Human*: expressed by circulating monocytes (21, 29, 41)	*Human*: highly expressed by circulating monocytes (21, 34, 41); differentially expressed on osteoclast precursors (42)	*Human*: expressed on circulating monocytes (21, 34) and classical, non-classical, and intermediate monocytes (31)	*Human*: expressed on majority of circulating monocytes, higher on CD16^−^ cells compared to CD16^+^ cells (23)

	*Mouse*: expressed by circulating monocytes (43)	*Mouse*: high expression of CD11b on murine monocytes (44)	*Mouse*: thought to be absent from most monocytes (45); though may be upregulated upon stimulation/maturation (44)	*Mouse*: lowly expressed by circulating monocytes, upregulated upon differentiation into macrophages (46), low ITGAD mRNA expression (25)

Macrophages	*Human*: expressed by monocyte-derived macrophages (21, 43); reduced expression on monocyte-derived macrophages compared to blood monocytes (21)	*Human*: expressed on monocyte-derived macrophages (47–49); expressed on alveolar macrophages, though at lower levels compared to blood monocytes (21)	*Human*: lowly expressed by monocyte-derived macrophages (21, 48–50)	*Human*: expressed on monocyte-derived macrophages *in vitro* (23)

	*Mouse*: expression dependent on tissue: present on pulmonary, but not on microglia, spleen or peritoneal macrophages (51)	*Mouse*: abundantly expressed by peritoneal macrophages (52, 53); highly expressed on dermal macrophages (54)	*Mouse*: expressed on alveolar macrophages (55); absent from bone marrow-derived macrophages and dermal macrophages (54)	*Mouse*: expressed by peritoneal macrophages (56)

Animal studies have been instrumental in elucidating integrin function in monocytes, macrophages, and DCs. β_2_ integrins are highly conserved across species, with mice, rats, and rabbits most commonly used as models. Importantly β_2_ integrin-deficient mice are considered an appropriate model of the human condition leukocyte adhesion deficiency (LAD) where β_2_ integrin expression or function is lost (57). However, while β_2_ integrin structure is largely similar between species, cellular expression levels can vary significantly. A common example is CD11c, which in mice is predominantly expressed by conventional (cDCs) and plasmacytoid DCs (pDCs), although can also be expressed on lymphocyte subsets. In humans, on the other hand, CD11c is expressed not only on DCs but also monocytes, macrophages, granulocytes, and natural killer cells (19, 38). Animal and human studies therefore have to be compared with great care, and validation of concepts conceived in animal models in human cells remains a priority in elucidating the functions of β_2_ integrins.

## β_2_ Integrins as Regulators of Immune Function

### Evidence for β_2_ Integrin Contribution to Immune Regulation

There is mounting evidence that puts β_2_ integrins at the center of the balance between immune priming and tolerance. Integrin-deficient humans and mouse models show that β_2_ integrins are important negative regulators of the immune system. LADs are genetic human disorders caused by the reduction or complete absence of β_2_-integrins (LAD-I) (58) or by mutations in the integrin-activating protein kindlin-3 (LAD-III) (59). These disorders are characterized by profound impairment of leukocyte recruitment to peripheral sites of infection. Patients with LAD suffer from increased susceptibility to infection and impaired inflammatory responses (60), resulting in markedly reduced lifespan if no therapeutic measures are taken. Paradoxically LAD patients also suffer from chronic inflammatory diseases. Examples of conditions prevalent in LAD patients include intestinal colitis (61) and periodontitis (62) suggest that β_2_ integrins have an important role in suppressing inflammation and promoting immune tolerance. Supporting this, the presence of functional β_2_ integrins improved symptoms in a model of skin inflammation by restricting DC-mediated T cell activation (63).

LAD pathology can be replicated in β_2_ integrin knockout (KO) mouse models, underlining the importance of β_2_ integrins for immune cell recruitment in both humans and murine models and the similarities between the species. From studies in KO mice and LAD patients, we know β_2_ integrins are essential in mediating T cell recruitment to lymph nodes and leukocyte, particularly neutrophil and T cell, recruitment to sites of inflammation. Here, we will further explore the roles of these integrins in monocytes, macrophages, and DCs.

### β_2_ Integrins Regulate Recruitment and Migration of Mononuclear Phagocytes

Evidence suggests that leukocyte recruitment *to* tissues is dependent on β_2_ integrins, because of the requirement for these adhesion molecules in the firm adhesion to the endothelial layer under shear flow conditions and for subsequent transendothelial migration (64). However, leukocyte migration *within* tissues is thought to occur independently of β_2_ integrins, as cells use an actin-dependent flowing and squeezing mechanism of movement in three-dimensional environments (64).

Geissmann and colleagues showed that the adhesion of patrolling murine monocytes to blood vessel walls is significantly decreased when CD11a is blocked (45). Similarly, chemotactic migration of human monocytes *in vitro* is inhibited when CD18 function is blocked (65). However, murine monocyte recruitment to sites of inflammation was found to occur independently of CD11a and CD11b (66), suggesting that β_2_ integrins are primarily involved in the homeostatic migration of monocytes and that their role is redundant during inflammation. On the other hand, increased expression levels of CD11d on macrophages mediates their retention at inflammatory sites in mice (56).

The role of β_2_ integrins in DC and macrophage recruitment to secondary lymphoid organs and tissues seems to be dependent on the inflammatory state of the body. Bone marrow-derived DCs (BMDCs) from mice where all integrins, including β_2_, are knocked out, migrated from the site of injection (ear) to the draining lymph node in similar numbers to their wild-type counterparts when activated with lipopolysaccharide (LPS). This suggests that DC migration during inflammation is not dependent on integrins. However, under steady-state conditions, the absence of functional β_2_ integrins from murine BMDCs (using signaling-deficient β_2_ integrin knock-in BMDCs) was found to increase migration from tissue (footpad) to draining lymph node, leading to the hypothesis that β_2_ integrins function to restrict migration in the steady-state by anchoring DCs in the tissue site. As a consequence of increased DC migration to the draining lymph node, the same study showed an increase in Th1 cytokine production (67), further supporting a negative regulatory role for β_2_ integrins on DCs. In addition, a murine model of skin inflammation also showed an increase in migratory DCs in the draining lymph node of β_2_ integrin signaling-deficient mice, as well as at the site of inflammation, though whether this was dependent on the inflammation or not was not determined (63). Overall, the cellular environment seems to determine the requirement for functional β_2_ integrins in the migration of both monocytes and DCs *in vivo*: integrins play a role in monocyte recruitment and DC migration under steady-state conditions, but are dispensable during inflammation.

### β_2_ Integrins Regulating DC–T Cell Interactions

In addition to their roles in leukocyte recruitment and migration, β_2_ integrins are also important mediators of cellular interactions. Functional β_2_ integrins are important in the formation of the immunological synapse between antigen-presenting cells (APCs) and T cells. The context and dynamics of this interaction determine whether T cells become activated or tolerized. β_2_ integrins, and their ligand, ICAM-1, are expressed by both the T cell and the APC and are vital in immune synapse formation. Importantly, it is becoming increasingly clear that β_2_ integrins expressed by the APC and T cell have opposing functions in the immune synapse, resulting in differential outcomes for the T cell response.

On the T cell side, CD11a clusters in the peripheral supramolecular activation cluster (P-SMAC) and binds to ICAM-1 on the APC (68). This molecular interaction stabilizes the connection made between T cell receptor and peptide:MHC on the APC in the central SMAC (16, 69), thereby enhancing TCR signal transduction (70). While T cell CD11a therefore has a largely pro-inflammatory effect, enhancing T cell activation, proliferation, and differentiation, a role for T cell integrins in regulation of activation, for example, in different T cell subsets, is not ruled out.

On the APC side of the immunological synapse, β_2_ integrins have also been shown to be involved, likely binding to ICAM-1 on the T cell. Importantly, the integrins on the APC regulate the outcome of the T cell response. For example, in murine models, active CD11b on DC surfaces inhibits the DC–T cell interaction (71). The reduced antigen-presenting capabilities of murine bone marrow-derived macrophages compared to BMDCs were therefore proposed to be due to their comparably larger surface expression of activated CD11b (71, 72). This suppressive role for DC CD11b has also been shown in human cells. When CD11b on human monocyte-derived DCs binds its ligand ICAM-1, both CD86 expression on DCs and DC-induced T cell proliferation were reduced (73). Interestingly, ligation of CD11b/CD18 decreases the ability of murine BMDCs to stimulate T cells and elicit a downstream response (74), CD11b/CD18 interactions can suppress Th17 cell differentiation (75), suggesting a strong role for this specific β_2_ integrin in immune regulation. This suggests that the activated conformation of CD11b/CD18 is extensively involved in regulating the immune system and has strong negative and positive regulatory functions depending on cell type they are expressed on.

Furthermore, the expression of activated β_2_ integrins on murine DC surfaces significantly reduces T cell activation (71) and further studies actually demonstrated an inverse relationship between forced activation of murine BMDC CD11a and T cell activation (72), suggesting a directly limiting effect of active β_2_ integrins on T cell activation by APCs.

Overall, the role of integrins as adhesion molecules carefully mediating and regulating cellular interactions is not to be underestimated for mounting an effective immune response.

### β_2_ Integrins Regulate Immune Cell Signaling

In addition to their roles in leukocyte recruitment and interactions, several studies show that integrin outside-in signaling following ligand binding can directly affect cell function. Chinese Hamster Ovarian cells transfected with CD11c acquire the ability to bind both LPS and Gram-negative bacteria, as well as the ability to initiate downstream activation signals (76). In contrast to their anti-inflammatory roles on DCs, CD11b or CD11c receptor occupation on the surface of human monocytes stimulates cell-specific pro-inflammatory pathways (77), such as secretion of IL-8, MIP1α, and MIP1β.

Generally, the interplay between TLR4- and β_2_ integrin-mediated signaling is controversial. On the one hand, it has been shown that CD11b positively regulates TLR4 signaling (78), especially in murine BMDCs. Several studies report β_2_ integrins act in synergy with LPS (79–81), therefore suggesting a potential pro-inflammatory role for CD11b. By contrast, other studies report that β_2_ integrins negatively affect TLR signaling. Complete absence of β_2_ integrins in mice (CD18 KO) was shown to result in a strong increase of TLR signaling (82) and the absence of CD11b specifically from murine macrophages causes exacerbated TLR-mediated inflammatory responses, resulting in increased susceptibility to endotoxin shock and *Escherichia coli* sepsis (83). Mechanistically, CD11b signaling has been shown to induce degradation of the key TLR signaling components, MyD88 and TRIF, directly dampening TLR responses in macrophages (83). Moreover, activation of CD11b on human inflammatory arthritis synovial macrophages *via* binding to its ligand ICAM was shown to indirectly inhibit TLR signaling (84) by inducing expression of IL-10 and the inhibitory factors SOCS3, ABIN-3, and A20. Integrins furthermore restrict TLR signaling on both murine macrophages and DCs (63). The role of β_2_ integrins in modulating TLR signaling is, therefore, complex, although one could tentatively propose that CD11b specifically seems to have opposing TLR4-mediated roles in inflammation, depending on the APC surface it is expressed on. However, while this could hold true for TLR4 signaling, this might not be the case for all TLRs. CD11b deficiency in murine BMDCs, while negatively affecting TLR4-mediated pathways, actually leads to an increase in DC cross-priming of cytotoxic T cells, a process mediated by the microRNA-146a (85). β_2_ integrin regulation of TLR-mediated responses therefore remains incompletely understood, with future studies hopefully elucidating the complex and intricate nature of these receptor interactions.

A variety of studies available suggest a significant immunoregulatory role for β_2_ integrins, not only by their mediation of adhesive and migratory processes, but also by immunological signaling. However, other studies suggest that, given the right cellular environment or cell type, β_2_ integrins can also have a strong pro-inflammatory effect (see Table 2 for comparison). When considering these opposing functions of integrins, it seems likely that even slight disturbances in integrin expression, signaling or activation could result in significant immunological effects, thus potentially contributing to a variety of autoimmune, inflammatory, and infectious conditions.

**Table 2:** Summary of the roles for β_2_ integrins in monocytes, macrophages, and dendritic cells (DCs).

Cell type	Recruitment and migration	Interactions with T cells	Signaling
Monocytes	β_2_ integrins mediate recruitment of monocytes under homeostatic conditions (45, 65), but dispensable for recruitment during inflammation (66)	Yet to be determined	Yet to be determined

	*Pro-inflammatory*	*Unknown*	*Unknown*

Macrophages	β_2_ integrins reported to mediate macrophage retention at inflammatory sites (56, 86)	Yet to be determined	β_2_ integrin signaling dampens macrophage responses to Toll-like receptor (TLR) stimulation (82, 83)

	*Pro-inflammatory*	*Unknown*	*Regulatory*

DCs	Under homeostatic conditions β_2_ integrins restrict DC migration from tissue to lymph nodes (67); Migration from tissue site to draining lymph nodes during inflammation occurs independently of integrins (64)	DC integrins contribute to contact formation with T cells—this role inhibits full T cell activation (71, 72, 74)	β_2_ integrin signaling functions to restrict DC activation both in response to TLR stimulation and under homeostatic conditions (67)

	*Regulatory*	*Regulatory*	*Regulatory*

## β_2_ Integrins in Inflammation, Infection, and Autoimmunity

Evidence for the role of β_2_ integrins in contributing to the development and progression of inflammatory and autoimmune conditions is accumulating. Considering that β_2_ integrin signaling can have opposing functions depending on subunit pairing and the immune cell type it is expressed on, it is not surprising that these receptors play important roles in both *contributing to* as well as *negatively regulating* inflammatory processes.

Human genetic studies point to a role of β_2_ integrins in inflammation and autoimmunity. A polymorphism of *ITGAM*, the CD11b subunit, increases the risk for the autoimmune disease systemic lupus erythematosus (87) (SLE), which shares genetic risk factors with rheumatoid arthritis (RA) (88). Disease risk for inflammatory bowel disease, similarly characterized by dysregulation of immune function specifically in the intestine, increases with amplified expression of alleles for both *ITGAL*, encoding CD11a, and the β_2_ integrin ligand *ICAM1* (89). Gene expression of CD11d in humans and mice was found to be increased in white adipose tissue in obesity, a condition characterized by an increase in systemic inflammation (90). Furthermore, CD11d activation led to increased IL-1β expression (23), which when overproduced can contribute to a variety of autoinflammatory conditions (91). While dysregulation of β_2_ integrin signaling seems likely to be involved in a variety of autoimmune diseases and inflammatory conditions, exact mechanisms are still unclear, and further investigation of both signaling pathways and genetic basis will be needed to fully elucidate their complex roles.

Recent studies have focused on β_2_ integrin involvement in RA, which serves as an excellent example of the opposing roles β_2_ integrins can take in disease. Expression of CD11a is increased in inflamed synovial tissue, where it is hypothesized to contribute to cell activation and on-going joint destruction (92, 93), but not in peripheral blood of RA patients. However, as CD11a is also involved in facilitating immune cell migration to sites of inflammation, clear-cut cause and effect of the presence of activated β_2_ integrins in the synovium is difficult to establish. Blocking all β_2_ integrins reduced inflammation in a rabbit RA model (94), while absence of CD11a led to complete resistance to disease induction in a KB × *N* serum transfer mouse model of arthritis (95). Furthermore, both a small molecule antagonist against CD11a and a CD11a-monoclonal antibody (mAb) proved to be similarly successful in reducing both inflammatory-mediated bone destruction and cytokine mRNA levels within the murine joint (96, 97). Mice with mutations in the β_2_ integrin ligand ICAM-1 also show reduced susceptibility to the collagen-induced arthritis (CIA) model (98). Clearly, CD11a–ICAM-1 interactions are essential for leukocyte recruitment to the inflamed joint.

However, evidence is emerging that other β_2_ integrins may function to control inflammation in arthritis. CD11b KO mice, for example, show exacerbated joint pathology in the KB x N serum transfer model of arthritis, underlining the starkly opposite roles different β_2_ integrins can play (95). A recent study replicated these results in a CIA model and, furthermore, showed that exacerbated joint pathology resulted from elevated IL-6 levels and an increase in Th17 cell priming, which could be rescued by introducing a CD11b-expressing DC cell line (99). On the other hand, blocking CD11b immediately before onset of disease significantly reduced disease burden in two different models of arthritis (CIA and a DBA/1 to severe combined immunodeficiency transfer model of arthritis) (100), suggesting that the role of CD11b in inflammatory arthritis may differ depending on the cell type involved and the disease stage.

When considering the importance, as well as the obvious complexity, of β_2_ integrin function in autoimmune diseases such as RA, therapeutically targeting β_2_ integrins will have to be carefully balanced but also holds great promise to offer novel treatment options.

## Applicability of Integrin-Targeting Therapies

Modulating integrin function to improve mal-adaptation or excessive activation of the immune system is of great interest in a variety of autoimmune and inflammatory conditions. However, achieving efficacy without immunocompromising side effects might prove challenging. Here, we discuss the progress and failures in developing integrin-targeted therapies and speculate on the routes forward for success.

To date, targeting integrins therapeutically has had mixed success in the clinic. The only mAb targeting β_2_ integrins, Efalizumab, which targets CD11a, was originally developed as a treatment for psoriasis (101). However, several patients presented with the potentially fatal disease progressive multifocal leukoencephalopathy (PML), caused by reactivation of the JC virus, which results in a white matter disorder of the brain (102). Although the mechanism of PML development in Efalizumab-treated patients was not investigated, we speculate that viral reactivation was likely either due to the loss of immune cell recruitment to the brain to control the virus (103) or due to the mAb itself crossing the blood–brain barrier (104). Due to the occurrence of PML, Efalizumab was withdrawn from European and American markets due to its associated safety issues in 2009.

Although targeting β_2_ integrins has so far failed in the clinic, targeting other integrins for the treatment of colitis and Crohn’s disease has proved successful. The mAb against the α_4_ integrin, Natalizumab, was developed for the treatment of multiple sclerosis and Crohn’s disease (105, 106). This mAb binds to α_4_β_1_ and α_4_β_7_. However, PML also occurs in some Natalizumab-treated patients (integrin α_4_β_1_ is also involved in leukocyte recruitment to the brain) and so is no longer used widely (107). More recently, a specific α_4_β_7_ targeting mAb Vedolizumab has shown success in safety efficacy in Crohn’s disease and ulcerative colitis. This success story underlines the potential of targeting integrins for therapeutic purposes.

In order to realize the potential of targeting β_2_ integrins therapeutically, it will be necessary to improve the strategy. As indicated by the success of Vedolizumab over Natalizumab, one way to do this is to target the right integrin subunit(s) in order to reduce the likelihood of side effects. Targeting CD11a, in the form of Efalizumab, proved unsuccessful in the clinic. As CD11a is expressed by almost all leukocytes, has vital roles in leukocyte recruitment and has immunoregulatory effects in mononuclear phagocytes, the resulting serious side effects from targeting this molecule therapeutically are, perhaps, not surprising. Targeting other CD11 subunits might be a more effective strategy. For example, CD11b, CD11c, and CD11d have a more restricted pattern of expression in leukocytes (predominantly on monocytes, macrophages, and DCs), which may make these molecules more suitable targets. Importantly, it is vital that we consider the pro- and anti-inflammatory functions of β_2_ integrin subunits and design drugs to target them appropriately. CD11b, for example, has clear regulatory roles in macrophages and DCs, meaning that we could potentially exploit this immuno-suppressive pathway by activating, rather than blocking, this integrin subunit. Such a strategy may have less risk of serious side effects. It is, therefore, essential that we fully understand the specific functions of individual integrin subunits in different leukocyte populations in order to target β_2_ integrin subunits effectively in the clinic.

Another option to explore is blocking not the β_2_ integrin itself, but the ligand of interest. Targeting the CD11a and CD11b ligand, ICAM-1, has shown beneficial results especially in early RA (108), although immunogenicity of the mAb in question restricts clinical use (109) and problems caused by impaired leukocyte recruitment prevail.

Further potential difficulties in developing integrin-targeting therapy include the close signaling relationships that exist in some integrins, potentially leading to complex downstream effects mediated even by an activating mAb highly specific for a β_2_ integrin (110). Carefully elucidating downstream signaling pathways and further increasing drug specificity is therefore essential to bring more integrin therapeutics into the clinic.

Innovative avenues to explore include computationally designed integrin proteins with constitutively activated or inactivated subunits, which could find applications in both pharmacological testing and therapy (111). Furthermore, developing small molecular drugs targeting β_2_ integrins viable for oral use remains a priority, as it could offer an alternative way to yield the same beneficial results without the dangerous side effects of mAbs. An example is the small molecule CD11b agonist, Leukadherin-1, which previous studies found to reduce monocyte-mediated TNF-release by mimicking natural ligand binding. When NK cells and monocytes were pre-treated with Leukadherin-1, innate inflammatory signaling in human *ex vivo* studies was suppressed (112). While the study noted some caveats, for example, the differences of CD11b function on different cell types (78), the drug is still being explored for the treatment of SLE. Another small molecule currently in development is the CD11a antagonist BMS-587101, which acts by reducing CD11a-mediated adhesion and to a lesser effect T cell proliferation. It significantly improved both murine models of lung inflammation and transplant viability (113).

Continuous effort to increase drug specificity and further understand their complex delicate signaling networks will be needed to bring β_2_ integrin-targeting drugs into the clinic. But while the use of integrin-targeting drugs has been contentious in the past, their potential in treating a wide variety of immune diseases is enormous and should not be neglected.

## Conclusion

This review explored the opposing nature of β_2_ integrin pro- and anti-inflammatory functions in three main immune functions, making them prime candidates to be both important mediators and regulators of the immune system. The first is migration, which allows for targeted immune cell recruitment to sites of infection and tissue damage. The second is adhesion, not only preceding immune cell extravasation at sites of inflammation, but also an important factor in initiating the adaptive immune response by facilitating cellular interactions. Finally, immune cell signaling, which allows for fine-tuned cooperation between a wide variety of immune cells. Considering the fact that β_2_ integrins play a complex role in three important areas of the immune system and their differential expression on monocytes, macrophages and DCs, it becomes clear that the variety of studies presented in this review is by no means exhaustive. The common message is evident: β_2_ integrins are involved in complex immunoregulatory signaling pathways. However, in addition to their well-established pro-inflammatory roles in recruitment and activation, β_2_ integrins also have essential immunoregulatory functions. Dysregulated integrin signaling, expression and surface activation is therefore likely to contribute to a variety of inflammatory and autoimmune conditions. Elucidating the function of β_2_ integrins further therefore promises to provide novel therapeutic targets for various disorders, RA being just one example.

## Author Contributions

CH and VM designed the structure of the review. LS wrote the first draft. CH and VM revised the manuscript. LS composed the figures. All authors have seen and agreed on the finally submitted version of the manuscript.

## Conflict of Interest Statement

The authors declare that the research was conducted in the absence of any commercial or financial relationships that could be construed as a potential conflict of interest. The handling Editor declared a shared affiliation, though no other collaboration, with the authors LS and CH.
